# Mega-oss and Mega-TCP versus Bio-Oss granules fixed by alginate gel for bone regeneration

**DOI:** 10.1038/s41405-020-0042-8

**Published:** 2020-08-11

**Authors:** Tong-Yue Wang, Shu-Lan Xu, Zhi-Ping Wang, Jin-Yuan Guo

**Affiliations:** 1 Center of Oral Implantology, Stomatological Hospital, Southern Medical University, Guangzhou, 510220 China; 2 Department of Oral and Maxillofacial Surgery, Stomatological Hospital, Southern Medical University, Guangzhou, 510220 China

**Keywords:** Dental biomaterials, Peri-implantitis

## Abstract

**Objectives/Aims:**

Bone graft materials are widely used at present because inadequate bone volume is usually found in implant patients. To determine the biocompatibility of a new grafting material, in vitro research is routinely performed before animal experiments and clinical testing. However, during in vitro experiments, bone material particles might move during testing, which could affect the accuracy of the results.

**Materials and Methods:**

To evaluate the biocompatibility of new bone substitutes, Mega-oss and Mega-TCP were compared with Bio-Oss using osteoblast cells and osteoclast cells fixed with alginate gel. Cell morphology, viability, bone resorption, alkaline phosphatase (ALP) activity, and staining were tested to compare the biocompatibility differences in the performance of Mega-oss, Mega-TCP, and Bio-Oss.

**Results:**

Cells spread better on Mega-oss and Mega-TCP than the round shape on Bio-Oss. The 3-(4,5-dimethyl-2-thiazolyl)-2,5-diphenyl-2-H-tetrazolium bromide (MTT) results showed that Mega-oss, Mega-TCP, and sodium alginate had good viability. Meanwhile, Mega-oss and Mega-TCP had the same osteoblast differentiation ability as Bio-Oss. The resorption rates of Mega-TCP and Mega-oss were higher than those of Bio-Oss (24.4%, 15.3%, and 3.3%, respectively).

**Conclusion:**

Mega-oss and Mega-TCP might be useful alternative bone graft materials compared with Bio-Oss. In addition, fixing the materials with sodium alginate gel could be a new method for in vitro bone material experiments.

## Introduction

In recent decades, dental implants have been used in partially or edentulous jaws for oral reconstruction as a routine treatment procedure.^1^ Having enough bone height and width is crucial for implantation. However, bone defects are a common clinical finding. Periodontal disease, gasification of the maxillary sinus, atrophy, and trauma may result in vertical, transverse, and sagittal bone volume shortage, which may make implant placement difficult for function and aesthetics.^1^

Bone graft is a common solution. According to the statistics, bone graft is required for 25% of dental implants.^2^ An ideal bone graft should have the properties of osteoconduction, osteoinduction, osteogenesis, and structural support. Currently, there are three broad categories of bone graft: autogenous bone, allograft, and bone graft substitutes.^3^ As the current gold standard material for bone substitution, autogenous bone is limited in the amount that can be harvested, and it may cause donor site morbidity, long hospitalization, and higher costs.^3^ Therefore, many new bone materials are being investigated as an alternative. Among these substitutes, Bio-Oss® is one of the earliest and leading products worldwide. This xenogeneic material can enhance bone regenerative in the bone defect areas. It has been a safe therapy in maxillofacial and periodontal osseous defects affirmatory over the past decades.^4^ However, some studies have shown that it has low cell viability and poor differentiation.^5–7^ After implantation, the bone particles degrade slowly through the patient’s metabolism.^8^ Bio-Oss® particles can still be observed many years after implantation.^9,10^

To determine the biocompatibility of a new grafting material, in vitro research is routinely performed before animal experiments and clinical tests. Cell culture systems can be utilized to test the ability of grafts to support cell adhesion, proliferation, and differentiation to validate the biocompatibility.^11^ In addition, it could also be used to evaluate bone remodeling, including the elimination of mineralized bone by osteoclasts and the formation of bone matrix by osteoblasts. However, during the in vitro experiment, small materials might move during experimental operation or float with media, which could affect the accuracy of results. Thus, sodium alginate, a nontoxic material that can be crosslinked from liquid to hydrogel, was used as a glue to fix the bone material.

In this study, we attempted to evaluate the biocompatibility of new bone substitute materials, Mega-oss and Mega-TCP, compared with Bio-Oss® using osteoblast cells and osteoclast cells. The market price of Mega-oss and Mega-TCP is lower than Bio-Oss®, and the source of Mega-TCP is easier than Bio-Oss®. If Mega-oss and Mega-TCP are similar to Bio-Oss® in biocompatibility, then we think that Mega-oss and Mega-TCP could be alternative materials for Bio-Oss®. For the first time, in our study, we used sodium alginate as a glue to fix the bone particles and prevent them from floating or moving.

## Materials and methods

### Cell culture and differentiation

MC3T3-E1 cells (ATCC, Manassas, VA, USA) were cultured in α-minimum essential medium (α-MEM; Gibco), supplemented with 10% (v/v) fetal bovine serum (FBS; Gibco), 1% penicillin, and in a humidified atmosphere at 37 °C under 5% (v/v) CO_2_. Differentiation was induced by additional adding 50 mg/ml ascorbic acid and 10 mM β-glycerophosphate after the cells had reached 90% confluence. The cell culture medium was changed every other day for 7 d.

Mouse RAW 264.7 cells (ATCC, Manassas, VA, USA) were cultured in Dulbecco’s Modified Eagle’s Medium (DMEM; Gibco) containing 10% FBS and 100 units penicillin/ml at 37 °C in 5% CO_2_ atmosphere. After the cells were cultured for 1 d, 1α,25(OH)_2_D_3_ (25 nM/ml; Sigma-Aldrich) was added to the culture medium. The cell culture medium containing 1α,25(OH)_2_D_3_ was changed every 2 d during the remaining 9 d.

### Sample preparation

Mega-oss (MEGAGEN IMPLANT, Korea) was composed of 60% cancellous bone and 40% cortical bone of allograft. The size of the Mega-oss material ranged from 0.40 to 0.71 mm; Mega-TCP (MEGAGEN IMPLANT, Korea) was 100% β-tricalcium phosphate. The size of the Mega-TCP was 0.60–1.00 mm (CGM); Bio-Oss (Geistlich Biomaterials, Switzerland) was derived from bovine bone, and the size was 0.25–1.00 mm.

Alginate hydrogels were made from 3 wt% solutions of sodium alginates (Junsei Chemical Co. Japan) in cell culture media. First, a 6 wt% solution was made with sodium alginate and double distilled water (DDW) and sterilized by autoclaving. Then, this solution was diluted with the same volume of cell culture medium for a final concentration of 3% alginate. Minimum alginate solution was added to the bottom of the well, which can cover the surface of the plate. Then, Mega-TCP, Mega-oss, and Bio-Oss particles were placed on the top of the alginate solution and fully cover the surface. Sterile 0.1 M CaCl_2_ was used to crosslink the alginate solution in order to fix the particles. After gelation, we removed the floating particles, and the culture media were used to replaced CaCl_2_ solution, then the Mega-oss gel, Mega-TCP gel, and Bio-Oss gel samples were stored at 4 °C for use.^12^

### The efficiency of sodium alginate gel

To test the efficiency of anti-floating of sodium alginate gel, we took Mega-oss gel, Mega-TCP gel, Bio-Oss gel samples, and 0.5 g particles of Mega-TCP, Mega-oss, Bio-Oss into culture dishes (60-mm diameter). We added 3 ml water in each dish, shook the particles to make them lay in a single layer. The floating particles were collected. Next, we dried and weighed these floating bone particles.

To test the efficiency of anti-movement of sodium alginate gel. First, we placed Mega-oss gel, Mega-TCP gel, Bio-Oss gel samples, and one layer of Mega-TCP, Mega-oss, Bio-Oss on 6-well plates. Next, we chose five random fields under the microscope, added water at the rate of 2 ml/min with 200 μl pipette, and took pictures before and after adding water. We overlapped the before and after photos (Beyond Compare, Scooter Software, Inc., Madison, WI), calculated the area of the different part (IPP 6.0, Media Cybernetics, USA), and then divide by the total area to get the movement rate.

### Scanning electron microscopy (SEM)

Morphology of the attached cells on the materials was evaluated using SEM after 7 d of culture. The MC3T3-E1 cells were seeded on Mega-oss gel, Mega-TCP gel, and Bio-Oss gel samples in 48-well tissue culture plates with 5 × 10^5^ cells/well. After 7 d of cultivation, the cell-seeded samples were washed with phosphate buffer solution (PBS) and then fixed in a 2.5% glutaraldehyde solution (Sigma-Aldrich) for 2.5 h at room temperature and rinsed twice with PBS. Lyophilization (Freeze Dryer, BioTron Inc., Seoul, Korea) was used for dehydration without shrinking the alginate. The samples were observed using a HITACHI S-3000N SEM (HITACH, Japan) at a voltage of 15 kV. Images were recorded.

### Cell proliferation and viability-MTT assay

Cell viability test of Mega-oss, Mega-TCP, and sodium alginate were assessed using a dimethylthiazol diphenyl tetrazolium bromide (MTT; AMERSCO, USA) assay. The Mega-TCP, Mega-oss, Bio-Oss, and alginate gel were soaked in α-MEM medium containing 10% FBS at the concentration of 0.2 g/mL and then slowly shaken for 1, 3, 7, and 10 d, respectively. MC3T3-E1 cells were seeded into 96-well plates at a density of 1 × 10^4^ cells/well and incubated for 24 h. Next, the culture medium was replaced by 100 μL of the extraction liquid, and the plates were incubated for another 24 h. Then, MTT (5 mg/ml) was added to each well, and the cells were incubated for 4 h at 37 °C. Then, the reaction solutions were removed, and 100 μl dimethylsulphoxide (DMSO) was added to each well. After being slowly shaken for 30 min, the optical density was measured at the wavelength of 570 nm using the EI READ 400 (Biochrom Ltd., England). The mean value of five readings for each sample was used for the final results.

### Alkaline phosphatase (ALP) staining and activity

In total, 5 × 10^5^ cells/well MC3T3-E1 cells were seeded into 48-well plate samples: Mega-oss gel, Mega-TCP gel, Bio-Oss gel, alginate gel alone, and only cells as a control group. The culture medium with 50 mg/ml ascorbic acid and 10 mM β-glycerophosphate was changed every other day for 7 d. After 7 d, the cells were washed with PBS twice, and fixed with iced 70% ethanol for 1 h at room temperature, and then they were washed twice with DDW. A BCIP/NBT (Sigma-Aldrich) staining working solution was added (200 μL/well). The plate was incubated at room temperature in the dark until the desired color developed. We take every image in the same condition. Then we analyzed the images using Image-Pro Plus image analysis software version 6.0 (IPP 6.0, Media Cybernetics, USA), and integral optical density (IOD) of BCIP/NBT staining were quantified.

The MC3T3-E1 cells were seeded into 96-well plate samples at a density of 4 × 10^4^ cells/well and were cultured with differentiation agents mentioned before for 7 d. An ALP assay kit (TAKARA BioInc., Shiga, Japan) was used, and the ALP activities were examined, according to the manufacturer’s instructions. The absorbance was measured at 405 nm using the EI READ 400 (Biochrom Ltd., Cambridge, UK).

### Tartrate-resistant acid phosphatase (TRAP) staining of RAW 264.7 cells

RAW 264.7 cells were seeded into 48-well plates at a density of 3 × 10^4^ cells/well. The RAW 264.7 cells were cultured with a medium containing 1α,25(OH)_2_D_3_ (Sigma-Aldrich) for 9 d.^13^ To confirm the generation of multinucleated osteoclast-like cells, the RAW 264.7 cells were stained for TRAP using the TRAP-staining kit (Sigma-Aldrich). Cells were fixed in a 2.5% glutaraldehyde solution for 2.5 h at room temperature and stained, according to the recommended protocols. TRAP-positive multinucleated (more than three nuclei) cells were examined under light microscopy and photographed. Multinucleated cells were counted in ten different visual fields (magnification × 200) for each well.

### Resorption pit assay of RAW 264.7 cells

RAW 264.7 cells were seeded on samples (Mega-oss gel, Mega-TCP gel, and Bio-Oss gel) at a density of 3 × 10^4^ cells/well in 48-well plates. After the 9 d of culturing with medium containing 1α,25(OH)_2_D_3_, the samples were sonicated in 0.1N NaOH for 2 min. The surface of each sample was visualized through SEM for evidence of resorption and quantitative analysis of the resorption area was performed with ImageJ software 1.44p.

### Statistical analysis

All numerical data were expressed as the means ± standard deviations (SD). Each experiment was measured twice. SPSS® for Windows, version 21.0 (SPSS Inc., Chicago, IL, US) was used for data analysis. Repeated measures analysis of variance, multivariate analysis of variance, and paired *t* test were used to assess the statistical significance of results. Differences were considered to be statistically significant at *p* < 0.05.

## Results

### Cell morphology

**Fig. 1:**
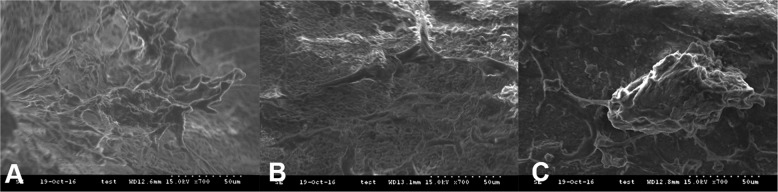
Scanning electron micrographs images of three particles seeded with cells after 7 d of cultivation. Scanning electron micrographs images of Mega-oss (**a**), Mega-TCP (**b**), and Bio-Oss (**c**) particles seeded with MC3T3-E1 cells after 7 d of cultivation, magnification × 700.

The scanning electron micrographs of Fig. 1a–c showed the adherence and growth of MC3T3-E1 cells. The appearance of the cells varied on different surfaces in their morphology, size, and density. The best result was observed on Mega-oss, the cells flat and spread well (Fig. 1a). On Mega-TCP, multiple MC3T3-E1 cells appeared as long and thin cellular extensions (Fig. 1b), whereas on Bio-Oss, separated and round cells with some stretching were observed (Fig. 1c).

### The efficiency of sodium alginate gel

We did not found any floating particle in Mega-oss gel, Mega-TCP gel, and Bio-Oss gel samples. The weight of floating particles in Mega-TCP, Mega-oss, and Bio-Oss was 0.047 ± 0.0051 g, 0.035 ± 0.0032 g, 0.045 ± 0.0039 g respectively. We calculated the floating rate of Mega-TCP, Mega-oss and Bio-Oss was 6.4%, 9.4%, and 8% respectively.

Mega-oss gel, Mega-TCP gel, and Bio-Oss gel samples did not move, that is the movement rate was 0%. The movement rate of Mega-TCP, Mega-oss, and Bio-Oss was 15.8 ± 1.9%, 12.73 ± 1.86%, and 13.8 ± 3.08% respectively.

### MTT

**Fig. 2:**
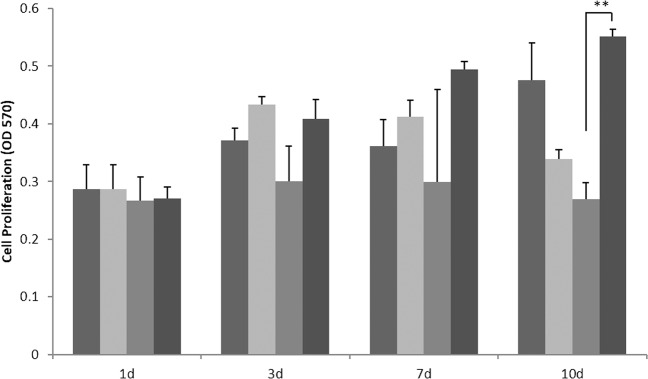
Cell proliferation of different bone materials and gel. Cell proliferation of MC3T3-E1 cells in extraction liquid soaked in Mega-oss, Mega-TCP, Bio-Oss, and sodium alginate for 1, 3, 7, and 10 d. The four columns (from left to right) are Mega-oss, Mega-TCP, Bio-Oss, and sodium alginate. Significant differences were assessed through repeated measures analysis of variance, multivariate analysis of variance and paired *t* test: **p < 0.01.

Cell viability/proliferation results were shown in Fig. 2. The OD values of the extraction liquid soaked in Mega-oss, Mega-TCP, Bio-Oss, and sodium alginate for 1, 3, 7, and 10 d were measured. On 1, 3, 7, and 10 d, the Mega-oss Mega-TCP and sodium alginate values were higher than that of the control group-Bio-Oss. However, only on 10 d, the sodium alginate was significantly higher than that of the Bio-Oss (*p* = 0.006). The cell proliferation of Bio-Oss did not change over time. The cell proliferation of Mega-oss and sodium alginate increased over time.

### ALP assay

**Fig. 3:**
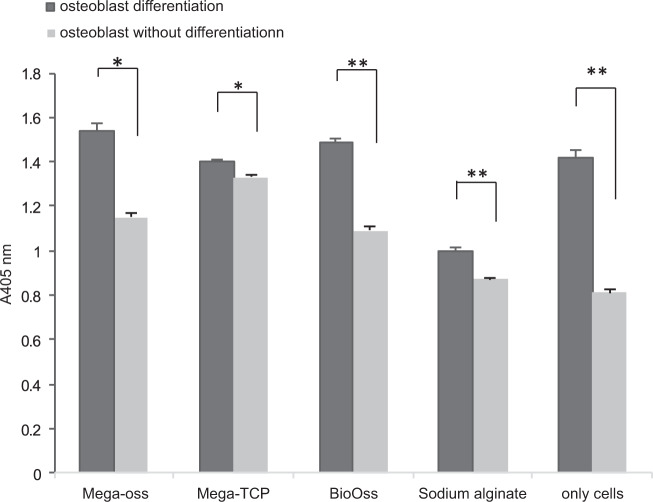
ALP activity of experimental groups and control groups. Alkaline phosphatase (ALP) activity of MC3T3-E1 cells grown on experimental groups (Mega-TCP, Mega-oss, and Bio-Oss) and control groups (sodium alginate and only cells). The left column means differentiation, and the right column means without differentiation in each group. Significant differences in all groups between differentiation and not differentiation. Significant differences were observed between differentiation of Mega-oss, Mega-TCP, and Bio-Oss with sodium alginate. Significant differences were assessed by a paired *t* test: *p < 0.05; **p < 0.01.

**Fig. 4:**
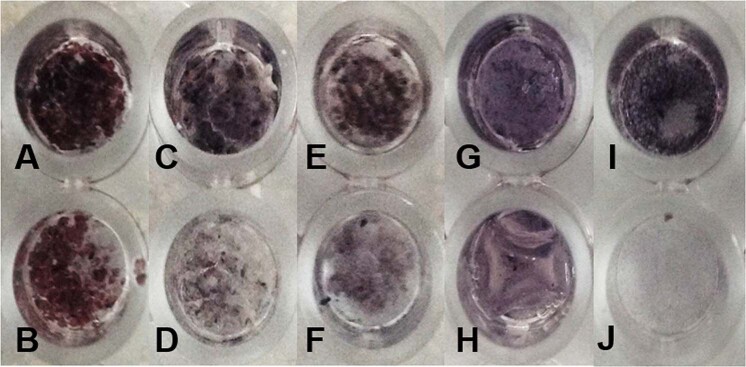
ALP staining in experimental groups and control groups. Alkaline phosphatase (ALP) staining of MC3T3-E1 cells in the experimental groups and control groups. MC3T3-E1 cells were cultured with or without differentiation for 7 d and stained with BCIP/NBT. **a** Mega-oss with differentiation; **b** Mega-oss without differentiation; **c** Mega-TCP with differentiation; **d** Mega-TCP without differentiation; **e** Bio-Oss with differentiation; **f** Bio-Oss without differentiation; **g** Sodium alginate with differentiation; **h** Sodium alginate without differentiation; **i** only cells with differentiation; **j** only cells without differentiation. BCIP/NBT, 5-bromo-4-chloro-3′-indolyphosphate p-toluidine salt/nitro-blue tetrazolium chloride.

**Fig. 5:**
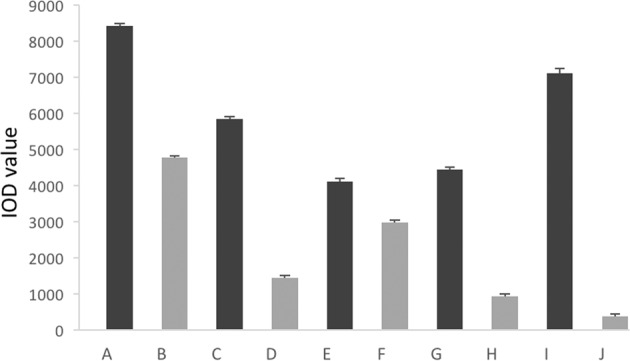
IOD value of ALP staining in experimental groups and control groups. IOD value of Alkaline phosphatase (ALP) staining of MC3T3-E1 cells. The integral optical density (IOD) of BCIP/NBT staining was quantified. **a** Mega-oss with differentiation; **b** Mega-oss without differentiation; **c** Mega-TCP with differentiation; **d** Mega-TCP without differentiation; **e** Bio-Oss with differentiation; **f** Bio-Oss without differentiation; **g** Sodium alginate with differentiation; **h** Sodium alginate without differentiation; **i** only cells with differentiation; **j** only cells without differentiation.

ALP activity was performed with MC3T3-E1 cells seeding on samples for 7 d in differentiation media, and the results are shown in Fig. 3. Significant differences were found between differentiation and non-differentiation in the Mega-TCP, Mega-oss, Bio-Oss, sodium alginate, and only cell groups. Statistical analysis indicated that there were no significant differences between Mega-TCP, Mega-oss, and Bio-Oss, although the Mega-oss had the highest OD value (1.54 ± 0.03), followed by Bio-Oss (1.49 ± 0.02) and Mega-TCP (1.40 ± 0.01). Mega-oss, Mega-TCP, and Bio-Oss showed significantly higher ALP activities compared with the control group: sodium alginate, *p* = 0.037, *p* = 0.001, and *p* = 0.027 respectively. ALP staining (Fig. 4) and its IOD of each well (Fig. 5) confirmed the ALP activity results. Positive samples of ALP staining were observed in the differentiation samples. In Fig. 5, the IOD values of A and B, C and D, E and F, G and H, I and J are significantly different. These mean that the osteoblasts differentiated on Mega-TCP, Mega-oss, Bio-Oss, sodium alginate, and only cell groups. Among the differentiated groups, there are significant differences statistically in comparison between any two groups. These results indicated that Mega-TCP, Mega-oss, and sodium alginate do not negatively influence osteoblast differentiation; Mega-TCP, Mega-oss, and Bio-Oss could promote osteoblast differentiation; Mega-TCP and Mega-oss have the same ability as Bio-Oss in osteoblast differentiation.

### TRAP staining

**Fig. 6:**
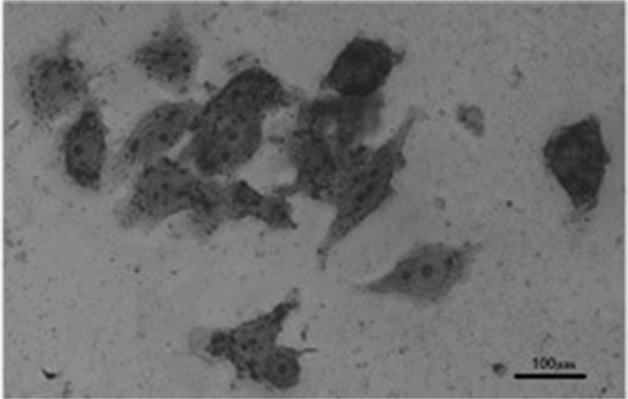
TRAP staining of differentiated RAW 264.7 cells. RAW 264.7 cells were cultured for 9 d in Dulbecco’s Modified Eagle’s Medium culture media with 1α,25(OH)2D3 (25 nM/ml). TRAP-positive multinucleated osteoclast-like cells were showed by TRAP staining. Light microscopy, magnification × 200. Scale bar: 100 μm.

**Fig. 7:**
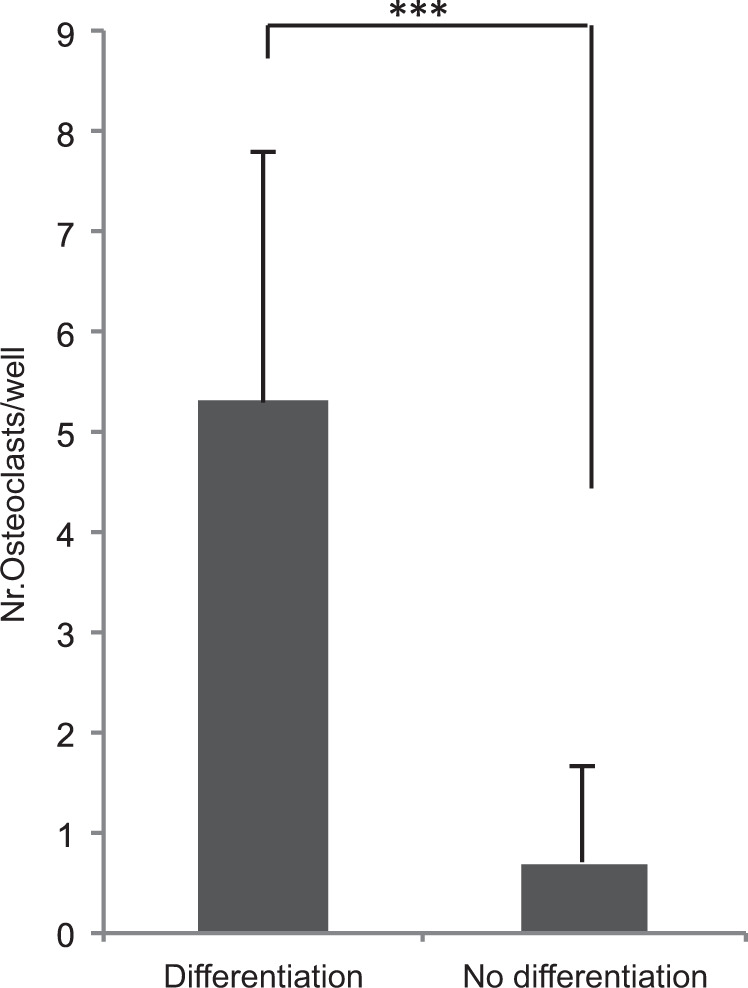
Multinucleated cell number of differentiated and non-differentiated groups. For 9 d, RAW 264.7 cells were cultured with and without medium containing 1α,25(OH)2D3. TRAP-positive multinucleated (more than three nuclei) cells were counted in ten different visual fields (magnification × 200) of each well through light microscopy. Significant differences were assessed by a paired *t* test: ***p < 0.001.

After culturing with medium containing 1α,25(OH)_2_D_3_ for 9 d, the TRAP-positive multinucleated osteoclast-like cells were confirmed by TRAP staining (Fig. 6). The RAW 264.7 cells treated with 1α,25(OH)_2_D_3_ showed a significantly higher number of TRAP-positive multinucleated cells than the control group (without 1α,25(OH)_2_D_3_). The TRAP-positive cell number in 96-well plate were 5.3 ± 2.49 cell/well and 0.70 ± 0.98 cell/well in the experiment group and control group, respectively (*p* < 0.001, Fig. 7). The results demonstrated the formation of osteoclast-like cells.

### Resorption pit assay

**Fig. 8:**
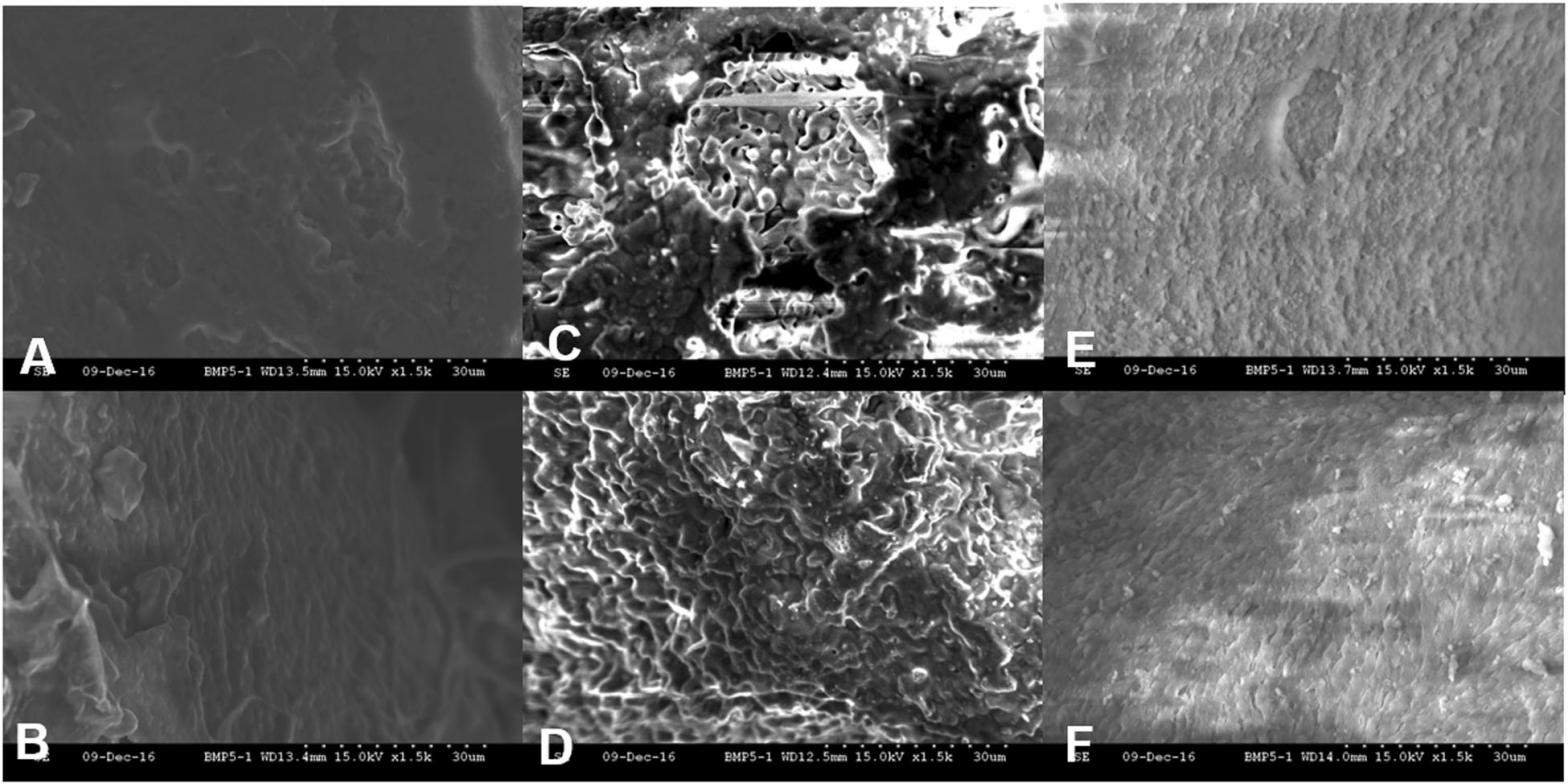
Bone resorption on different bone materials. **a** Mega-oss with osteoclast differentiation; **b** Mega-oss without osteoclast differentiation; **c** Mega-TCP with osteoclast differentiation; **d** Mega-TCP without osteoclast differentiation; **e** Bio-Oss with osteoclast differentiation; **f** Bio-Oss without osteoclast differentiation.

After removing cells on the materials, resorption areas are shown in Fig. 8. In Table 1, all the three materials showed significant differences in the resorption area between with and without 1α,25(OH)_2_D_3_ differentiation (*p* = 0.015 in the Mega-oss group, *p* = 0.005 in the Mega-TCP group and *p* = 0.004 in the Bio-Oss group). Resorption pits on Mega-TCP surface were most noticed with 24.36 ± 20.57% resorption area, followed by Mega-oss and Bio-Oss (15.31 ± 16.07% and 3.28 ± 1.90%, respectively). Compared with the control group (Bio-Oss group), the area of resorption was significantly increased in the Mega-TCP and Mega-oss groups, with *p* = 0.04 and *p* = 0.011 (Table 1). There were no significant differences between the Mega-TCP group and Mega-oss groups.

**Table 1:** The resorption areas on different bone materials.

	Osteoclast differentiation	Without differentiation	*p* value
Mega-oss (%)	15.31 ± 16.07*	0.18 ± 0.48	0.015
Mega-TCP (%)	24.36 ± 20.57*	1.47 ± 2.41	0.005
Bio-Oss (%)	3.28 ± 1.90	0.70 ± 0.83	0.004

The areas of resorption on different bone materials were measured using ImageJ software. Significant differences were found in the Mega-oss, Mega-TCP, and Bio-Oss groups between RAW 264.7 cells, with and without differentiation. The resorption areas in the Mega-oss and Mega-TCP groups were higher than those in the Bio-Oss groups. Data are shown as means ± SD. Significant differences were assessed with a paired *t* test: *p < 0.05.

## Discussion

In our study, we developed a new in vitro bone graft material test model for basic science research, in which the bone particles were fixed by sodium alginate gel. In the preexperiment, we found that the size of particles ranged from 0.25 to 1.00 mm; therefore, some of them have less density than water. Thus, the bone particles will move or float when changing the media, no matter how careful and slow the action. In case of the graft flowing or moving affecting the results, we used the glue to fix the particles on the bottom. Sodium alginate is found in marine life (brown algae), with a linear anionic natural polysaccharide. It has many favorable features, such as biodegradability, nontoxicity, gelling in situ, and low costs.^14^ It can be crosslinked by Ca^2+^, forming an alginate hydrogel. With these attractive features, it has been used in many biomedical applications, including cell transplantation, graft scaffold, wound healing, and drug delivery.^12,15,16^ Our study result also proved the good biocompatibility of sodium alginate. The cell amount increased with the time pass by and on 10 d was the highest value. The cell viability was higher than Bio-Oss at all time points, and the value was even twice that of Bio-Oss at 10 d. The sodium alginate gel will not affect the test results of the bone materials. With this model, we used the alginate to cover the bottom of each well, then bone materials were spread on the alginate. The floating rate of Mega-TCP, Mega-oss, and Bio-Oss are 6.4%, 9.4%, and 8% respectively, while the floating rate of Mega-oss gel, Mega-TCP gel, and Bio-Oss gel was all 0%. And the movement rate of Mega-TCP, Mega-oss, and Bio-Oss was 15.8 ± 1.9%, 12.73 ± 1.86%, and 13.8 ± 3.08% respectively. Mega-oss gel, Mega-TCP gel, and Bio-Oss gel samples did not move at all. This is because the bone-gel complex was crosslinked together, thus, the bone could not move. The height of alginate was not higher than half of the bone. Therefore, the results are not affected and more reliable. In addition, if the bone materials implanted in body, adding gels could help ossify faster without movement.

For the evaluation of a new bone graft materials, in vitro experiments usually occur before animal experiment or clinical testing.^17^ The prerequisite for a bone graft material to gain bone regeneration is the attachment of osteoblasts or their precursors and their proliferation on the surface. The Mega-oss in our study belongs to allografts. Papadopoulos et al.^18^ reported that the allografts did not affect the proliferation of the tested cells. Osteoblasts seeded onto allograft and Bio-Oss showed a homogeneous distribution pattern, good adhesion, and pericellular deposition of extracellular matrix, observed by SEM. In 1 week, osseous matrix adhered to the grafts in each group. Three weeks later, intimate contact with osteoblasts embedded in a mineralized, fibril-rich extracellular matrix was observed in both groups. There were no significant differences in cell viability or proliferation.^19^ In our study, cells stretched well on the allografts, compared with round shape on Bio-Oss. In addition, the cell amount of Mega-oss grew from 1, 3, 7 to 10 d. At these four time points, Mega-oss showed higher OD values than Bio-Oss, which means that the cells proliferated better on Mega-oss than Bio-Oss.

Mega-TCP in our study is composed of β-TCP, and the properties are highly similar to the inorganic properties of human bone. According to Bernhardt et al. the macroporosity of β-TCP enhances bone ingrowth, which will facilitate bone formation and resorption.^17^ β-TCP has shown good biocompatibility. The morphology and roughness of the surface affect the attachment and differentiation of the osteoblasts and may also enhance cell spreading. According to Sammons et al.^20^ the relatively smooth surfaces of the Bio-Oss granules do not support cellular attachment. Cerasorb M® is a synthetic bone graft from phase pure β -TCP. According to Bernhardt et al.^17^ the cell numbers on Cerasorb M® are significantly higher compared with the numbers on Bio-Oss at 7, 14, and 28 d of cultivation. In our study, on Mega-TCP, multiple long and thin cells were observed, while on Bio-Oss, single and round cells were observed (Fig. 1). However, compared with Mega-TCP, Mega-oss exhibited better attachment (at the attachment area) and extracellular matrix secretion. Mega-oss and Mega-TCP showed better proliferation than Bio-Oss. And no cytotoxicity of Mega-oss and Mega-TCP was found compared with Bio-Oss (Fig. 2), as the OD values were all higher than those of Bio-Oss.

Bone is a highly dynamic organ by a remodeling process to renew itself. The bone remodeling process consists of four consecutive stages: bone resorption, in this step osteoclasts gnaw old bone; reversal, during this time, mononuclear cells attach to the bone surface; bone formation, in which osteoblasts completely replace resorbed bone with new bone; and bone mineralization, during this stage, osteocytes are embedded within the bone matrix.^21^ Thus, the abilities of bone forming and resorption are important properties for bone materials. Orti et al.^22^ showed that the regenerated bone of allografts was similar to the original bone in terms of histological structure. Kubosch et al.^19^ reported that the expression of osteogenic biomarkers of human osteoblasts cultured on allograft and Bio-Oss were similar. Ayobian-Markazi et al.^23^ showed that Bio-Oss suppresses the proliferation and differentiation of osteoblasts. The ALP activity of Cerasorb was higher than that of Bio-Oss. Bernhardt et al.^17^ reported cell number on Bio-Oss samples was too low to test the ALP activity. In the present work, Mega-TCP, Mega-oss, and Bio-Oss could all promote osteoblast differentiation (Figs. 3, 4, 5). As Mega-oss, Mega-TCP and Bio-Oss showed significantly higher ALP activities compared with the control group. Among the differentiated groups (Fig. 5), there are significant differences statistically in comparison between any two groups. Mega-TCP and Mega-oss has a better ability than Bio-Oss in osteoblast differentiation. The only cell group had high value is because the surface of alginate is smooth which is not suitable for cell adhesion. The difference of these studies and ours is probably due to the different methods and the number of seeding cells.

The ideal tissue engineering materials should be resorbed and replaced in line with the newly regenerated biological tissue of the patient’s own body.^24^ β-TCP has shown good biocompatibility, osteoconduction, and resorption properties. It can gradually be resorbed and eventually replaced by new bone.^25,26^ Previous studies have reported that the surface layers of TCP-ceramic enhanced bonding with adjacent host bone. This stimulates osteoclastic resorption and osteoblastic new bone formation within the resorbed implant.^27^ Immunohistochemical results indicated that after 14 d of culture the differentiated osteoclast-like cells were well attached to the surface of TCP before initiating resorption.^28^ After 4 weeks of implantation, the area fraction of newly formed bone was significantly increased in amorphous TCP-containing PLGA fibers, compared with neat polymer electrospun scaffolds. The PLGA/TCP-treated defects developed to a spongiosa bone, while a solid cortical bone-like appearance was observed in defects treated with Bio-Oss. Bio-Oss consisted of bovine bone mineral which may cause difficulties to distinguish from newly formed bone and Bio-Oss, during direct comparison between bone regeneration of treated defects with PLGA/TCP and Bio-Oss.^29^ The resorption ability and capacity of simultaneously supporting new bone formation has been shown in animal models and human trials. It can gradually be resorbed and eventually replaced by new bone.^25,26^ In our study, we did the bone forming and resorption experiment at the same time. The results reported in Fig. 7 shows that the resorption rate of Mega-TCP was higher than that of Mega-oss, and the resorption rate of Mega-oss was higher than that of Bio-Oss, with resorption areas 24.4%, 15.3%, and 3.3%, respectively. The resorption area in Mega-TCP group was ~5 times greater than Bio-Oss, while the resorption area in Mega-oss group was nearly 3 times greater than Bio-Oss. As we have previously mentioned, the resorption rate of Bio-Oss is slow. The resorption rate of Mega-oss is close to that of in vivo bone. However, our results showed a much higher resorption rate for Mega-TCP. Augmentation with fast resorbable TCP ceramics may lead to a loss of bone mass.^17^ Kurashina et al. found that after 100% porous β-TCP was implanted in rabbits, β-TCP was obviously degraded, with no bone formed.^30^ However, the resorption characteristics of TCP depend on the porous structure and the mineral composition as well as the degree of sintering.^26^

This study has several limitations. Testing the changes in cells at the genetic level would yield further information. Further animal and clinical studies are necessary to confirm these findings.

## Conclusions

The in vitro experimental results demonstrate that Mega-oss and Mega-TCP have favorable effects on cell adhesion, proliferation, and osteoblastic and osteoclastic differentiation. From a tissue engineering perspective, a bone material that possesses these abilities is highly desirable. Thus, the novel Mega-oss and Mega-TCP might be useful alternative bone materials compared with the already established Bio-Oss. And animal and clinical experiments will be carried out in future study to testify this conclusion. In addition, it was shown for the first time that sodium alginate gel can serve as a graft fix gel. This finding could be a new method for in vitro experiments on bone materials.
